# Kinetic investigations and stability studies of two *Bothrops* L-amino acid oxidases

**DOI:** 10.1186/s40409-018-0172-9

**Published:** 2018-12-04

**Authors:** Tássia R. Costa, Sante E. I. Carone, Luiz F. F. Tucci, Danilo L. Menaldo, Nathalia G. Rosa-Garzon, Hamilton Cabral, Suely V. Sampaio

**Affiliations:** 1 Departamento de Análises Clínicas, Toxicológicas e Bromatológicas, Faculdade de Ciências Farmacêuticas de Ribeirão Preto, Universidade de São Paulo (FCFRP-USP), Avenida do Café, s/n, B. Monte Alegre, Ribeirão Preto, SP CEP 14040-903 Brazil; 2 Departamento de Ciências Farmacêuticas, Faculdade de Ciências Farmacêuticas de Ribeirão Preto, Universidade de São Paulo (FCFRP-USP), Ribeirão Preto, SP Brazil

**Keywords:** Snake venom, *Bothrops*, L-amino acid oxidase, Enzymatic stability

## Abstract

**Background:**

L-amino acid oxidases isolated from snake venoms (SV-LAAOs) are enzymes that have great therapeutic potential and are currently being investigated as tools for developing new strategies to treat various diseases, including cancer and bacterial infections. The main objective of this study was to make a brief evaluation of the enzymatic stability of two *Bothrops* LAAOs, one isolated from *Bothrops jararacussu* (BjussuLAAO-II) and the other from *Bothrops moojeni* (BmooLAAO-I) venoms.

**Methods and results:**

The enzymatic activity and stability of both LAAOs were evaluated by microplate colorimetric assays, for which BjussuLAAO-II and BmooLAAO-I were incubated with different L-amino acid substrates, in the presence of different ions, and at different pH ranges and temperatures. BjussuLAAO-II and BmooLAAO-I demonstrated higher affinity for hydrophobic amino acids, such as Phe and Leu. The two enzymes showed high enzymatic activity in a wide temperature range, from 25 to 75 °C, and presented optimum pH around 7.0. Additionally, Zn^2+^, Al^3+^, Cu^2+^ and Ni^2+^ ions negatively modulated the enzymatic activity of both LAAOs. As to stability, BjussuLAAO-II and BmooLAAO-I showed high enzymatic activity for 42 days stored at 4 °C in neutral pH solution. Moreover, the glycan portions of both LAAOs were analyzed by capillary electrophoresis, which revealed that BjussuLAAO-II presented two main glycan portions with relative masses of 7.78 and 8.13 CGU, while BmooLAAO-I showed three portions of 7.58, 7.94 and 8.37 CGU.

**Conclusions:**

Our results showed that, when stored properly, BjussuLAAO-II and BmooLAAO-I present enzymatic stability over a long time period, which is very important to allow the use of these enzymes in pharmacological studies of great impact in the medical field.

## Background

L-amino acid oxidases (LAAOs) are flavoenzymes found in different organisms; however, those from snake venoms (SV-LAAOs) are the most well-characterized enzymes of this protein family [1–3]. In recent decades, many studies have been dedicated to exploring the physical-chemical properties, structural characteristics and biological functions of SV-LAAOs from different snake species [3–7]. Such studies indicate that SV-LAAOs from different sources can present significant variances in molecular mass, substrate specificity, stability, enzymatic and biological activity [1, 8, 9].

SV-LAAOs are classified as FAD-dependent oxidoreductases and are capable of catalyzing the stereospecific oxidative deamination of L-amino acid substrates in α-keto acids. The catalytic cycle begins with the reductive half-reaction involving the conversion of FAD to FADH_2_ and concomitant oxidation of the amino acid to an imino acid. Then, this imino acid undergoes non-enzymatic hydrolysis, releasing α-keto acid and ammonia. An oxidative half-reaction completes the cycle by reoxidizing FADH_2_, thus producing hydrogen peroxide (H_2_O_2_) [9, 10]. Studies indicate that the numerous biological and pharmacological effects presented by SV-LAAOs are due, at least partially, to the H_2_O_2_ generated during their enzymatic reaction, because in the presence of catalase, an agent that degrades H_2_O_2_, such activities are generally inhibited [1].

The enzymatic activity of SV-LAAOs is determined by a horseradish peroxidase assay, where the H_2_O_2_ generated by their enzymatic reaction is consumed by the peroxidase to oxidize an o-phenylenediamine (OPD) substrate, generating a cationic radical that is monitored in a spectrophotometer between 380 and 492 nm. L-leucine is the substrate commonly used in this method, because LAAOs exhibit a preference for hydrophobic amino acids, such as leucine, phenylalanine and methionine [1, 9, 11, 12].

Most SV-LAAOs are thermolabile enzymes and may also be inactivated by variations in the pH of the storage buffer. Data from the literature suggest that SV-LAAOs should be stored at 4 °C in neutral pH buffers in order to avoid protein inactivation [1, 8, 13, 14]. Nevertheless, there are also reports of SV-LAAOs that are more stable despite thermal and pH variations, such as CR-LAAO from *Calloselasma rhodostoma* snake venom, which presents an alkaline optimum pH but remains stable in buffer solutions with neutral pH and temperatures ranging from 4 to 25 °C for many days or at − 20 °C for up to 60 h, in addition to maintaining its enzymatic activity even after lyophilization [11].

SV-LAAOs isolated from *Bothrops* snakes have been well studied in recent years, mainly in terms of their pharmacological effects such as antitumor and microbicide activities [5, 14–17]. BmooLAAO-I, for example, is a 65 kDa LAAO isolated from *Bothrops moojeni* venom, which exhibited antitumor, bactericidal and trypanocidal effects [16]. Another potential enzyme is BjussuLAAO-II, an acidic 60 kDa LAAO from *B. jararacussu* venom that exerted antiprotozoal effects against *Leishmania amazonensis* and *Trypanosoma cruzi* and also induced cytotoxicity and apoptosis in MCF7 tumor cells [5].

Therefore, although SV-LAAOs present great biotechnological applicability in the development of new drugs, the fact that they are unstable enzymes ends up being a limitation in the advancement of this research line. With that in mind, the present study describes a brief evaluation of the enzymatic activity of two *Bothrops* LAAOs (BmooLAAO-I and BjussuLAAO-II) in the face of variations in temperature, pH, interference of different ions and determination of kinetic parameters, as well as the characterization of the glycan portions of these proteins.

## Materials and methods

### Venoms and reagents

*B. jararacussu* and *B. moojeni* snake venoms were donated by The Center for the Study of Venoms and Venomous Animals (CEVAP) from São Paulo State University (UNESP), Botucatu, São Paulo, Brazil, and stored at − 20 °C. Other materials and equipment used were described throughout the methodology. Unless otherwise specified, reagents were of analytical grade.

### Purification of L-amino acid oxidases

BjussuLAAO-II was isolated from *Bothrops jararacussu* venom as described by Carone et al. [5], whereas BmooLAAO-I was purified from *Bothrops moojeni* venom according to Stábeli et al. [16].

### Determination of protein concentration

Protein concentrations were determined by the bicinchoninic acid assay using the Pierce™ BCA Protein Assay Kit (Thermo Fischer Scientific), according to the manufacturer’s instructions. Bovine serum albumin was used as a standard (Bio-Rad, Hercules, CA, USA).

### Determination of LAAO activity

LAAO activity was determined using the method described by Bordon et al. [3], with modifications. BjussuLAAO-II or BmooLAAO-I (2 μg) were incubated for 30 min, at room temperature, with 2 mM o-phenylenediamine (Sigma-Aldrich) prepared in methanol, 1 U/mL horseradish peroxidase (Sigma-Aldrich), 5 mM L-leucine (Sigma-Aldrich), and 0.1 M Tris-HCl buffer pH 7.2. The reaction was stopped with 100 μL of 10% citric acid (*v*/v) and absorbance was recorded at 490 nm.

The absorbance values were used to calculate the LAAO-specific activity in U/mg/min, which is the amount of H_2_O_2_ (μmol) formed per minute per mg of protein. The amount of H_2_O_2_ formed was quantified from a standard curve expressed in nmol/min, and the results were expressed as percentages of relative activity.

### Amino acid affinities for BjussuLAAO-II or BmooLAAO-I

LAAO activity was determined using the method described by Kishimoto and Takahashi [18] with modifications. BjussuLAAO-II or BmooLAAO-I were incubated with 2 mM o-phenylenediamine (Sigma-Aldrich) prepared in methanol (OPD), 1 U/mL horseradish peroxidase (Sigma-Aldrich), 5 mM of amino acids and 0.1 M Tris-HCl buffer pH 7.2. The reactions were incubated for 25 min, at room temperature, and were stopped with 100 μL of 10% citric acid (*v*/v); then, the absorbance was recorded at 490 nm.

To assess the affinity of both LAAOs on different substrates, the following L-amino acids were used at 5 mM: methionine (Met), isoleucine (Ile), phenylalanine (Phe), tyrosine (Tyr), histidine (His), glutamine (Gln), arginine (Arg), glycine (Gly), threonine (Thr), asparagine (Asn), valine (Val), alanine (Ala), aspartate (Asp), lysine (Lys), serine (Ser), glutamate (Glu), proline (Pro) and cysteine (Cys).

### Biochemical characterization of BjussuLAAO-II and BmooLAAO-I

The effects of pH and temperature variation on the enzymatic activity of both LAAOs were determined by a microplate assay: BjussuLAAO-II or BmooLAAO-I (2 μg) was incubated with 2 mM OPD, 1 U/mL horseradish peroxidase (Sigma-Aldrich) and 5 mM of L-leucine. For the evaluation of the effect of different pH values, 50 μL of the different buffers were added: acetate (pH 4.0, 4.5 and 5.0), MES (pH 5.5, 6.0 and 6.5), HEPES (pH 7.0, 7.5 and 8.0), Bicine (pH 8.5 and 9.0) and CAPS (pH 9.5 and 10.0), all at 0.1 M, followed by incubation for 20 min at room temperature.

The effects of temperature on the LAAO activity was investigated in the range of 25 °C to 80 °C, by incubating BjussuLAAO-II or BmooLAAO-I (2 μg) with 2 mM OPD, 1 U/mL horseradish peroxidase (Sigma-Aldrich), 5 mM of L-leucine and 0.1 M HEPES buffer pH 7.

The effects of different ions on the enzymatic activity of BjussuLAAO-II and BmooLAAO-I was determined in the presence of the following salts: aluminum chloride (AICI_3_), barium chloride (BaCl_2_), calcium chloride (CaCl_2_), cobalt chloride (CoCl_2_), copper chloride (CuCl_2_), lithium chloride (LiCl), magnesium chloride (MgCl_2_), manganese chloride (MnCl_2_), potassium chloride (KCl), sodium chloride (NaCl), nickel sulfate (NiSO_4_) and zinc sulfate (ZnSO_4_). BjussuLAAO-II or BmooLAAO-I (0.1 μg) were previously incubated with ions at the final concentration of 5 mM for 5 min at 45 °C. Then, 2 mM OPD, 1 U/mL horseradish peroxidase (Sigma-Aldrich), 5 mM of L-leucine and 0.1 M HEPES buffer pH 7 were added. The enzymatic reaction was incubated for 12 min at 45 °C.

All reactions were stopped with 100 μL of 10% citric acid (*v*/v) and the absorbances of the reaction mixtures were measured on a SpectraMax Spectrophotometer Microplate Reader (Molecular Devices) at 490 nm. The results were expressed as percentage of relative activity.

### Determination of LAAO kinetic parameters

The kinetic parameters K_m_, k_cat_ and k_cat_/K_m_ of BjussuLAAO-II and BmooLAAO-I were calculated by the non-linear adjustment of the experimental data by the Michaelis and Menten equation using the software GraphPad Prism 5.0. The enzymatic activity of LAAOs was determined using as substrates: leucine, methionine, isoleucine, phenylalanine, tyrosine, histidine and glutamine, in increasing concentrations from 0.25 mM to 5 mM. BjussuLAAO-II or BmooLAAO-I (2 μg) was incubated with 2 mM OPD, 1 U/mL horseradish peroxidase (Sigma-Aldrich) and 0.1 M HEPES buffer pH 7. Reactions were incubated for 12 min at 45 °C and the absorbance of the reaction mixtures was measured at 490 nm.

### Assessment of the stability of stored LAAOs

The enzymatic activity of BjussuLAAO-II and BmooLAAO-I was monitored for 42 days from their respective purifications. Samples from both LAAOs were aliquoted and stored at different temperatures: 4 °C and − 20 °C. In addition, we evaluated the enzymatic activity of samples that were lyophilized or heated at 100 °C. The assays were carried out according to the LAAO activity assay previously described (item 2.4), expressing the results as percentages of relative activity. The enzyme was protected from light under all the tested conditions.

### Analysis of the glycan portions by capillary electrophoresis

The glycan portions of BjussuLAAO-II and BmooLAAO-I were analyzed using the LabChip GXII Touch equipment, preparing 150 μg of each protein with the ProfilerPro Glycan kit (PerkinElmer cat. 760,525). The proteins were submitted to a process of deglycosylation with PNGase F, which cleaves their N-linked carbohydrates. After fluorescent labeling, the glycans were separated by microchip capillary electrophoresis; next, the generated electropherogram was analyzed together with a pattern composed of a mixture of glucose oligomers in the analysis program LabChip GX Reviewer. The electropherogram is presented as a graph of CGU (Caliper Glucose Units) by fluorescence intensity. Samples and pattern are aligned by the addition of a lower marker (peak with 6.60 CGU), which allows overlapping electropherograms to compare them.

### Statistical analysis

The results were presented as mean values ± SD (*n* = 3). Statistical analyses were performed by the software GraphPad Prism 5, using one-way ANOVA method with Tukey’s post-test, considering values of *p* < 0.05 to be significant.

## Results and discussion

**Fig. 1:**
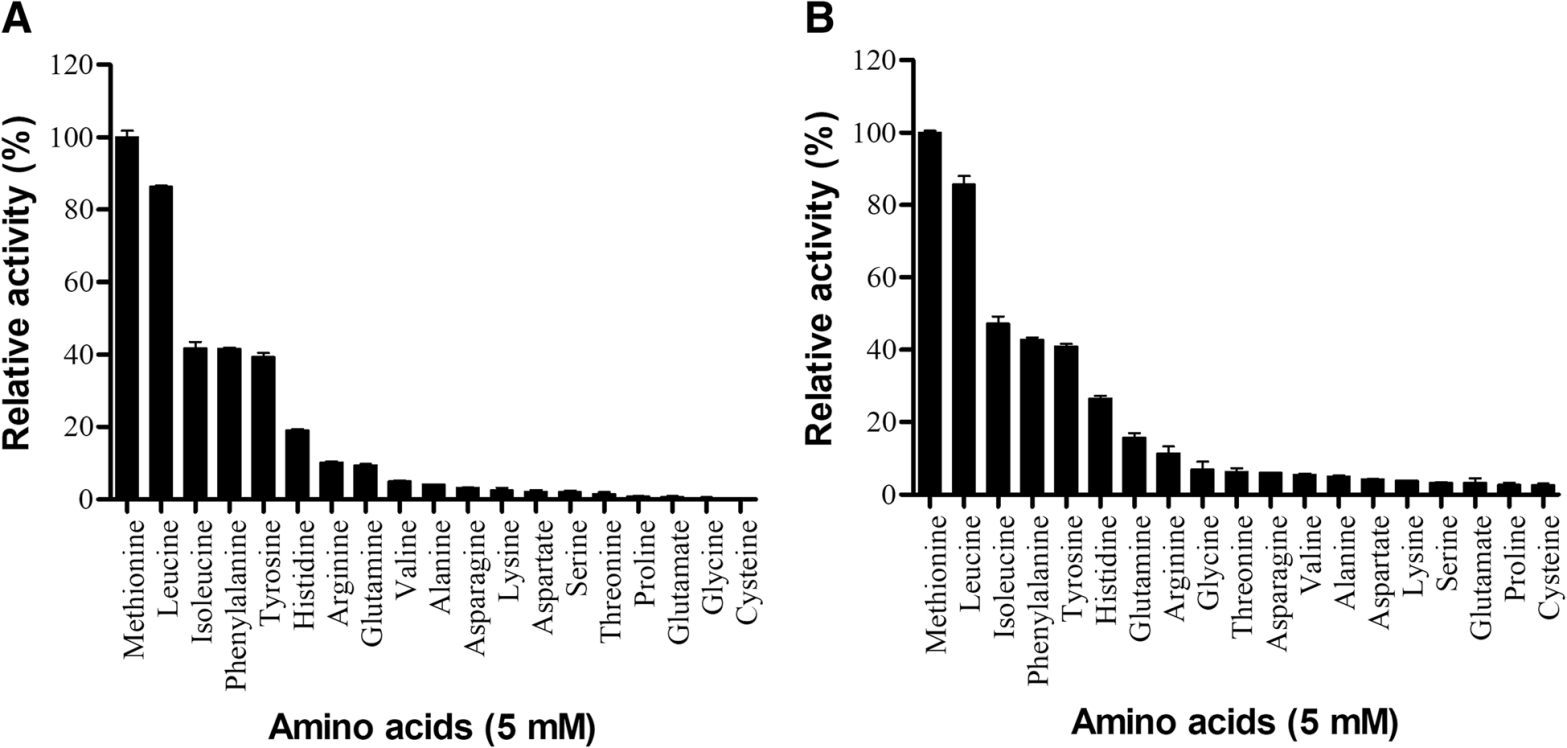
Evaluation of the specificity of BjussuLAAO-II (**a**) and BmooLAAO-I (**b**) action on different L-amino acid substrates. LAAO activity was determined by incubating each LAAO (2 μg) for 25 min at room temperature with 2 mM o-phenylenediamine, 1 U/mL horseradish peroxidase, 5 mM of each L-substrate and 0.1 M Tris-HCl buffer pH 7.2. The reaction was stopped with 10% citric acid and absorbance was recorded at 490 nm. The results were then expressed as percentages of relative activity, considering the highest activity to be 100%. Data expressed as mean values ± SD (*n* = 3)

BjussuLAAO-II and BmooLAAO-I displayed broad enzymatic activity on different L-amino acids (Fig. 1). According to our results, both LAAOs demonstrated the following order of amino acid specificity: Met > Leu > Ile > Phe > Tyr > His, with low or absent catalytic activity on most of the other amino acids evaluated (Fig. 1). Kinetic studies suggest that SV-LAAOs show specificity for hydrophobic and aromatic amino acids, while their affinity for polar, basic and acidic amino acids is lower. This can be explained by the fact that LAAO substrate-binding sites comprise three hydrophobic subsites, presenting one or two methyl/methylene carbons, and an amino-binding subsite [19, 20]. Thus, positively charged amino acids such as L-lysine and L-arginine exhibit unfavorable electrostatic interactions with the catalytic site of the enzyme [9, 11, 12]. Our results for BjussuLAAO-II and BmooLAAO-I corroborate the data found for other SV-LAAOs [14, 15, 20–24], indicating that the catalytic site of these enzymes is conserved among different snake species.

We also investigated some kinetic parameters such as K_m_ and k_cat_ and calculated the k_cat_/K_m_ ratio to evaluate the affinity of each of the enzymes on a given substrate and also their catalytic efficiency ratio. BjussuLAAO-II showed higher specificity for Phe with 33,277 mM^− 1^.s^− 1^, followed by Leu and Met, with 23,500 and 16,661 mM^− 1^.s^− 1^, respectively (Table 1). BmooLAAO-I presented higher specificity for Leu, with 44,326 mM^− 1^.s^− 1^, followed by Phe and Met with 32,787 and 32,645 mM^− 1^.s^− 1^, respectively (Table 2). Both LAAOs showed lower affinity for Gln and His, as shown by K_cat_/K_m_ values below 1000 mM^− 1^.s^− 1^ (Tables 1 and 2).

**Table 1:** Kinetic parameters for BjussuLAAO-II

Amino acid	K_m_ (mM)	k_cat_ (s^−1^)	k_cat_/K_m_ (mM^−1^.s^− 1^)
	Mean ± SD	Mean ± SD	Mean ± SD
Phe	0.1 ± 0.0	3850 ± 18	33,277 ± 4061
Leu	0.3 ± 0.0	5755 ± 54	23,500 ± 952
Met	0.6 ± 0.1	10,200 ± 596	16,661 ± 1434
Tyr	1.1 ± 0.1	2740 ± 116	2549 ± 199
Ile	2.0 ± 0.1	2472 ± 39	1222 ± 30
His	14.1 ± 1.9	4935 ± 492	352 ± 14
Gln	3.2 ± 0.3	614 ± 10	192 ± 15

**Table 2:** Kinetic parameters for BmooLAAO-I

Amino acid	K_m_ (mM)	k_cat_ (s^−1^)	k_cat_/K_m_ (mM^−1^.s^−1^)
	Mean ± SD	Mean ± SD	Mean ± SD
Leu	0.2 ± 0.0	6688 ± 111	44,326 ± 3562
Phe	0.2 ± 0.0	4885 ± 78	32,787 ± 1859
Met	0.3 ± 0.0	9546 ± 270	32,645 ± 1952
Tyr	1.1 ± 0.1	4312 ± 212	3949 ± 353
Ile	1.4 ± 0.2	4110 ± 214	3072 ± 248
His	7.4 ± 0.7	5320 ± 412	722 ± 18
Gln	6.1 ± 0.5	1723 ± 95	285 ± 8

The oxidation of L-amino acids catalyzed by SV-LAAOs follows Michaelis-Menten kinetics. The kinetic parameters K_m_ and K_cat_ are useful for the study and comparison of different enzymes in relation to their substrate. Each enzyme presents optimum K_m_ and K_cat_ values that reflect the cellular environment, substrate concentration and chemical characteristics of the catalyzed reaction [9].

L-Leu is the most common substrate for SV-LAAOs; however, high catalytic constants (K_cat_/K_m_) have also been described for L-Phe, L-Met and L-Ile [1]. According to the results obtained by Abdelkafi-Koubaa and collaborators [4], the LAAO from *Cerastes cerastes* venom presented the highest specificity for Phe, followed by Met and Leu. Similarly, the LAAO from *Bothrops pirajai* venom also presented the highest specificity for Phe, followed by Tyr and Trp [25]. These results were similar to those obtained for BjussuLAAO-II, which also showed higher specificity for Phe. On the other hand, SV-LAAOs from *Crotalus durissus cumanensis* [26], *Lachesis muta* [27], *Daboia russelis* [28] and *Bothrops leucurus* [29] presented greater specificity for Leu, similarly to BmooLAAO-I.

In general, SV-LAAOs are highly sensitive to changes in temperature and pH; therefore, the usual suggestion is that these enzymes should be stored in neutral pH solutions at 4 °C [1]. Studies indicate that SV-LAAOs may remain active for variable periods of time over a wide temperature range (0 to 50 °C), with exposure to temperatures above 55 °C resulting in a gradual decrease in activity caused by disruptions in hydrophobic interactions and hydrogen bonds between different subunits of the enzyme [7, 9, 11, 15, 23, 24].

**Fig. 2:**
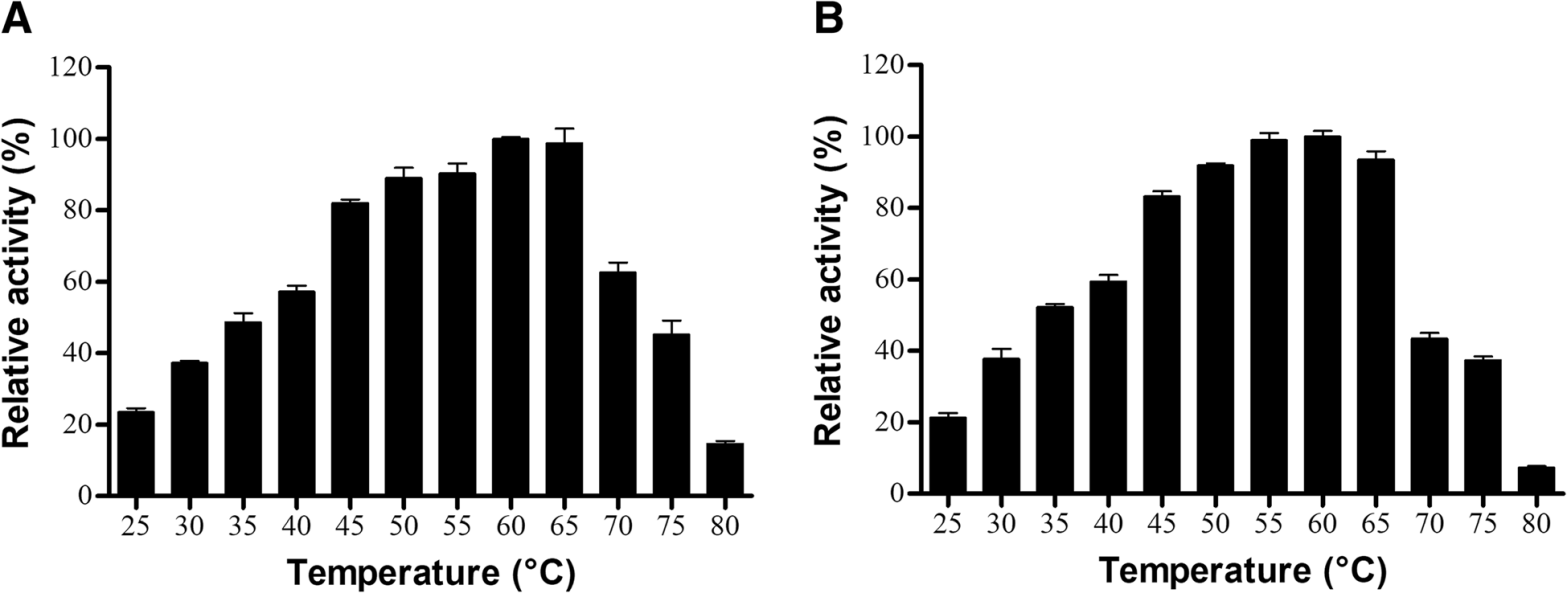
Effects of temperature variations on the enzymatic activity of BjussuLAAO-II (**a**) and BmooLAAO-I (**b**). LAAO activity was determined by incubating each LAAO (2 μg) for 10 min at temperatures from 25 to 80°C with 2 mM o-phenylenediamine, 1 U/mL horseradish peroxidase, 5 mM L-leucine and 0.1 M HEPES buffer pH 7.0 buffer pH 7.0. The reaction was stopped with 10% citric acid and absorbance was recorded at 490 nm. The results were then expressed as percentages of relative activity, considering the highest activity to be 100%. Data expressed as mean values ± SD (*n* = 3)

The apparent optimum temperature estimated for BjussuLAAO-II was 65 °C (Fig. 2a), and for BmooLAAO-I was 60 °C (Fig. 2b). However, it is possible to observe a broad spectrum of enzymatic activity by both *Bothrops* LAAOs, varying from 25 to 75 °C (Fig. 2). These data allow us to suggest that the target enzymes of our studies are more resistant to high temperatures than most of the SV-LAAOs already described.

**Fig. 3:**
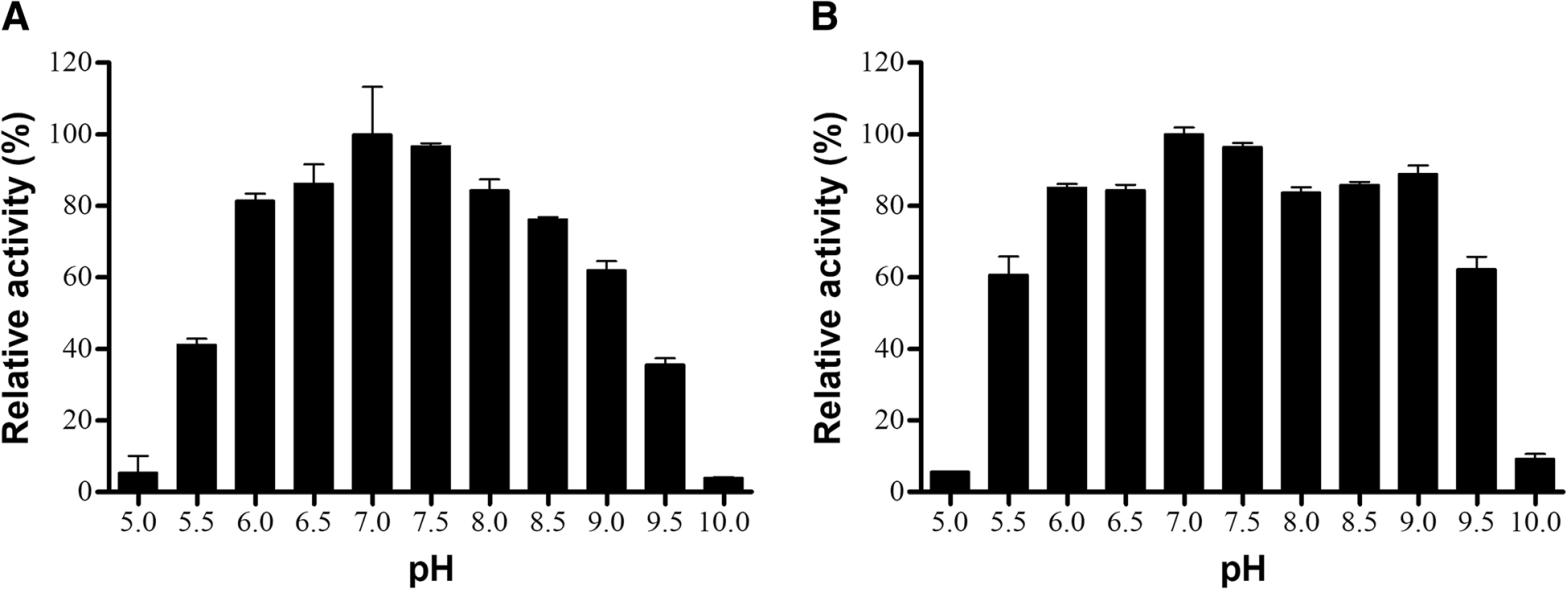
Effects of pH variations on the enzymatic activity of BjussuLAAO-II (**a**) and BmooLAAO-I (**b**). LAAO activity was determined by incubating each LAAO (2 μg) for 20 min at room temperature with 2 mM o-phenylenediamine, 1 U/mL horseradish peroxidase, 5 mM L-leucine and different pH buffers at 0.1 M. The reaction was stopped with 10% citric acid and absorbance was recorded at 490 nm. The results were then expressed as percentages of relative activity, considering the highest activity to be 100%. Data expressed as mean values ± SD (*n* = 3)

To identify the apparent optimum pH of the LAAOs from *B. jararacussu* and *B. moojeni* venoms, these enzymes were exposed to different pH ranges. The highest activity was presented by both LAAOs at a pH around 7 (Fig. 3); however, it was possible to observe a wide range with high activity by the two enzymes, mainly between pH 6.0 and 9.0 by BjussuLAAO-II (Fig. 3a) and from 5.5 to 9.5 by BmooLAAO-I (Fig. 3b). This is consistent with most of the SV-LAAOs described so far, which are active at pH values ranging from 5.5 to 9 [3, 7, 11, 27, 30].

**Fig. 4:**
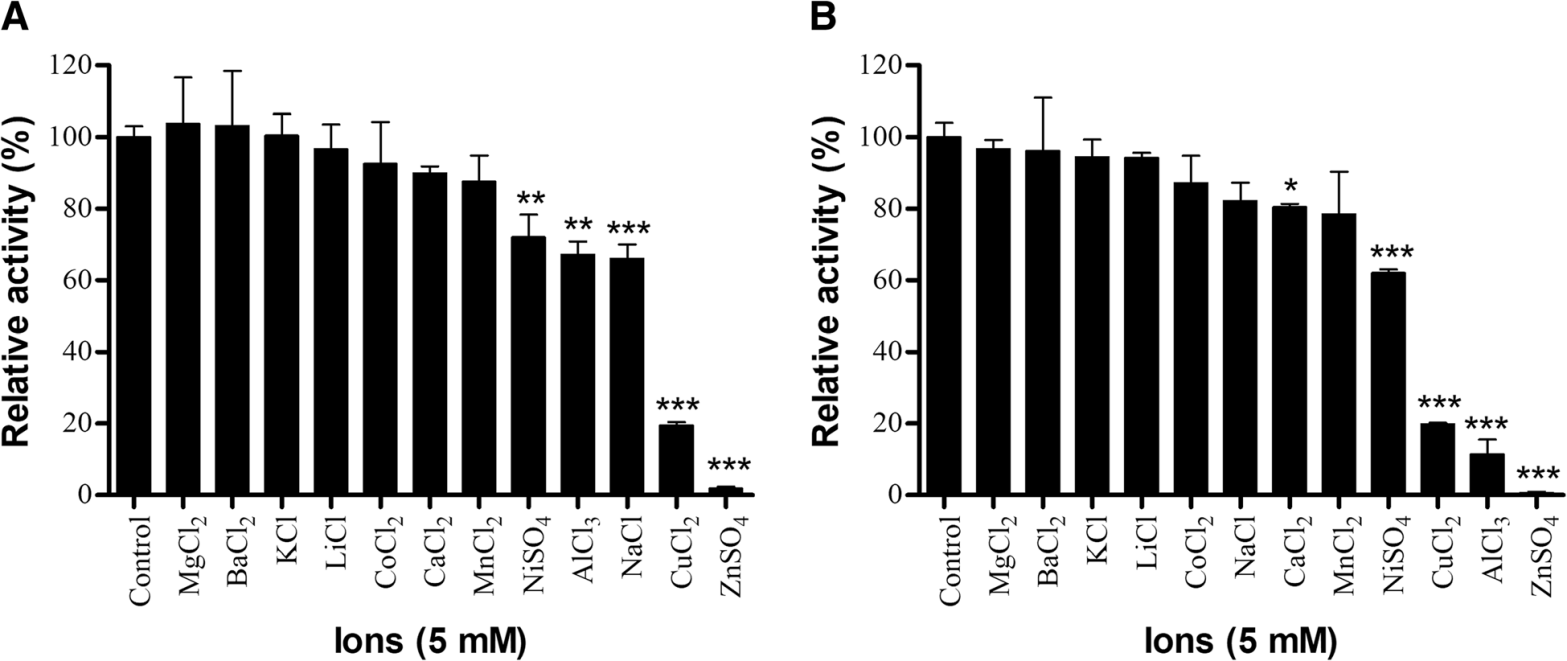
Effects of different ions on the enzymatic activity of BjussuLAAO-II (**a**) and BmooLAAO-I (**b**). LAAO activity was determined by incubating each LAAO (2 μg) for 12 min at 45°C with 2 mM o-phenylenediamine, 1 U/mL horseradish peroxidase, 5 mM L-leucine, 0.1 M HEPES buffer pH 7 and different salt solutions at the final concentration of 5 mM. The reaction was stopped with 10% citric acid and absorbance was recorded at 490 nm. The results were then expressed as percentages of relative activity, defining the control activity (without salts) as 100%. Data expressed as mean values ± SD (*n* = 3). Values significantly different from the control were indicated by *(*p*<0.05), **(*p*<0.01) or ***(*p*<0.001)

Another biochemical characteristic we identified in both *Bothrops* LAAOs was the effects that some ions exert on their enzymatic activity. These effects can be positive modulations when the activities of the enzymes increase in the presence of certain ions, or the opposite, i.e. negative modulations, when some ions decrease the enzymatic activity. Both LAAOs showed a significant reduction in their enzymatic activities when exposed to Zn^2+^, Al^3+^, Cu^2+^ or Ni^2+^ salts (Fig. 4). BjussuLAAO-II also had its activity affected in the presence of Na^+^ (Fig. 4a) and BmooLAAO-I in the presence of Ca^2+^ (Fig. 4b).

Different bivalent ions can activate or inhibit the specific activity of SV-LAAOs. The LAAO from *Crotalus adamantus* requires Mg^2+^, whereas those from *Lachesis muta* and *Bothrops brazili* were inhibited in the presence of Zn^2+^. Other ions such as Mn^2+^ and Ca^2+^ did not affect the activity of these LAAOs. The inhibitory action of these ions may be related to their ability to reversibly bind to thiol groups of cysteines present at the active site of the enzymes, reducing their activity and compromising their pharmacological effects [9].

BjussuLAAO-II and BmooLAAO-I also had their enzymatic activities monitored under different storage conditions. After isolation, aliquots of both LAAOs underwent lyophilization and completely lost their activities (results not shown). The same effect was observed in samples heated at 100 °C and samples stored for 7 days at − 20 °C (results not shown). SV-LAAOs are progressively inactivated when subjected to freezing or lyophilization [9, 15, 23, 27]. The inactivation by freezing (and also by changes in pH, as mentioned above) induces conformational changes in SV-LAAOs that can be demonstrated by circular dichroism [30], which may involve alterations in the enzyme binding, as well as in the affinity of the FAD cofactor for electrons [9]. Nevertheless, although SV-LAAOs can easily lose their enzymatic activities, it is also known that some inactive forms of SV-LAAOs can be reactivated, with studies showing that the most favorable reactivation conditions for that involve the treatment of the enzymes at 37 °C and pH ranging from 5.5 to 7.5 [1, 9, 30, 31].

**Fig. 5:**
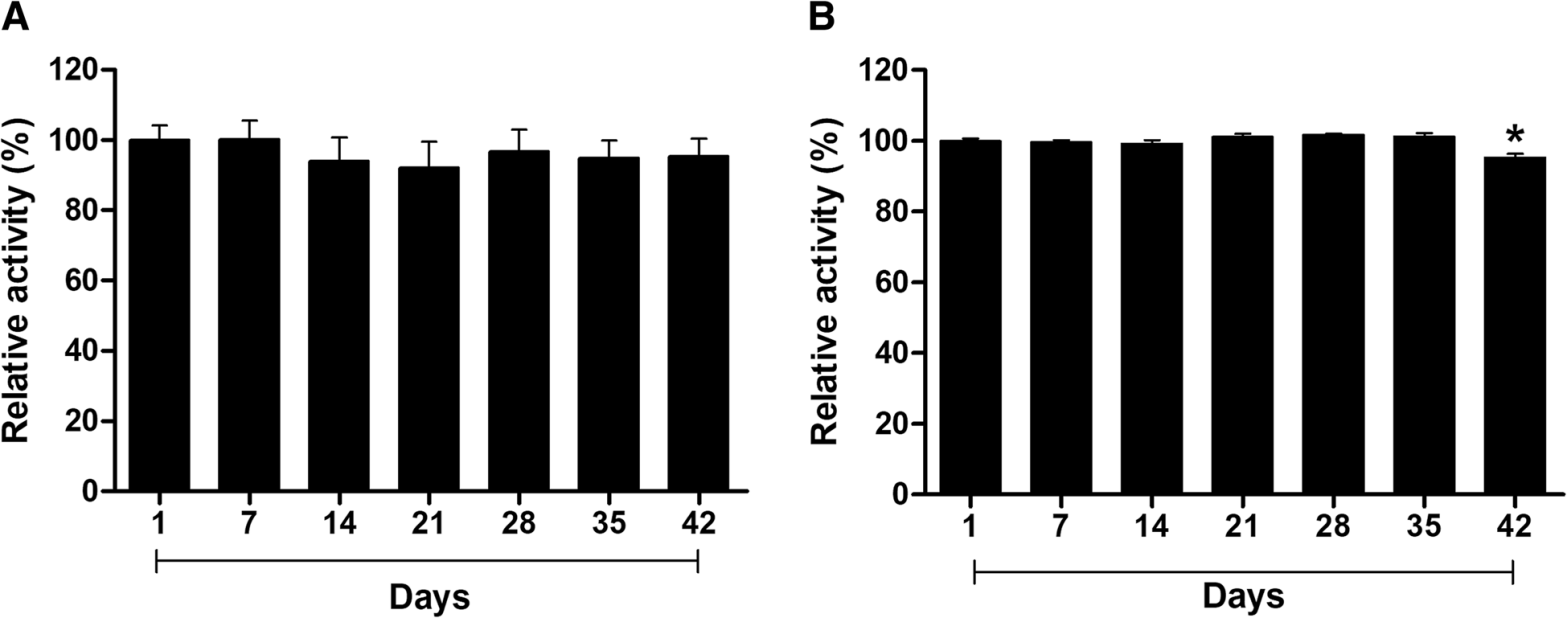
Evaluation of the enzymatic stability of BjussuLAAO-II (**a**) and BmooLAAO-I (**b**) over a 42-day period stored at 4ºC. LAAO activity was determined by incubating each LAAO (2 μg) for 30 min at room temperature with 2 mM o-phenylenediamine, 1 U/mL horseradish peroxidase, 5 mM of each L-substrate and 1 M Tris-HCl buffer pH 7.2. The reaction was stopped with 10% citric acid and absorbance was recorded at 490 nm. The results were then expressed as percentage of relative activity, defining the activity of day 1 as 100%. Data expressed as mean values ± SD (*n* = 3). Values significantly different from day 1 were indicated by *(*p*<0.05)

Both *Bothrops* LAAOs were also stored at 4 °C and evaluated for their enzymatic activity every 7 days for 42 days. At this temperature, BjussuLAAO-II maintained its maximum relative enzymatic activity for 42 days (Fig. 5a). BmooLAAO-I also maintained its high enzymatic activity during that storage period at 4 °C, only showing a small decrease in its activity (about 5%) on day 42 (Fig. 5b). Thus, our results corroborate data from the literature demonstrating that the enzymatic activity of SV-LAAOs is susceptible to changes in temperature, but we can also see that when stored properly (4 °C, pH 7), BjussuLAAO-II and BmooLAAO-I are stable over a long time period, which facilitates the use of these enzymes in pharmacological studies of great impact in the medical field.

In general, SV-LAAOs are homodimeric enzymes with molecular mass ranging from 120 to 150 kDa in the native form and from 50 to 70 kDa in their monomer forms. This variation in the molecular mass between different SV-LAAOs may be related to glycosylation sites, since these enzymes are usually glycoproteins [1, 8]. This class of enzymes is characterized by a variable percentage of glycans depending on the snake species [9, 11, 14, 16, 32], whereas carbohydrates such as fucose, mannose, galactose, N-acetylglucosamine and sialic acid have already been identified in the structure of these enzymes [33, 34]. These glycans are bound to the enzyme by N-glycosidic bonds and probably modulate their physicochemical properties, thus increasing the solubility and viscosity of the proteins and maintaining the stability of the electric charges [9, 16, 35].

**Fig. 6:**
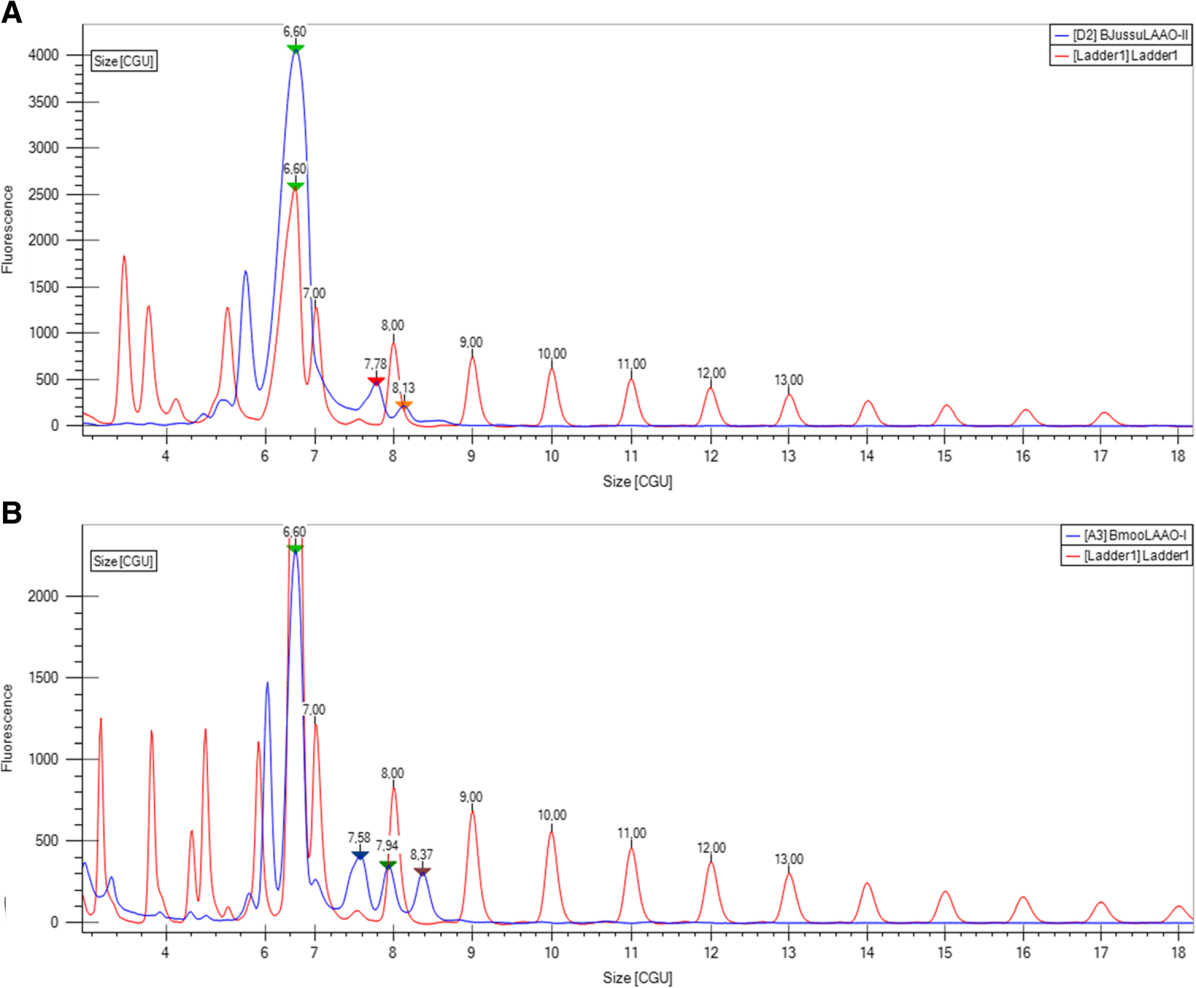
Evaluation of the glycan portions of BjussuLAAO-II (**a**) and BmooLAAO-I (**b**) by capillary electrophoresis. LAAOs were deglycosylated with PNGase F, and then glycans went through fluorescent labeling followed by separation by microchip capillary electrophoresis. The generated electropherogram was analyzed together with a pattern composed of a mixture of glucose oligomers, which allows the results to be presented as CGU (Caliper Glucose Units) by fluorescence intensity. Glycosylation profiles of LAAOs and pattern are shown in blue and red, respectively. Arrowheads indicate glycan peaks for the lower marker (6.60 CGU) and enzymes (7.78 and 8.13 CGU for BjussuLAAO-II; 7.58, 7.94 and 8.37 CGU for BmooLAAO-I)

In the electropherogram of the glycosylation profile of BjussuLAAO-II (Fig. 6a), we can observe the presence of two major peaks, with relative masses between 7 and 9 glucose monomers. This finding indicates that the glycan portion of BjussuLAAO-II is composed of 2 species of oligomers with masses of 7.78 and 8.13 CGU. In the electropherogram of the glycosylation profile of BmooLAAO-I (Fig. 6b), there are 3 major peaks, with relative masses of 7.58, 7.94 and 8.37 CGU.

One of the most interesting characteristics that SV-LAAOs present is the number of glycosylation sites, sharing an N-glycosylation consensus sequence in the C-terminal portion (Asn361) [36–38]. This glycan portion in the C-terminal region seems to be related to some biological activities of SV-LAAOs. Studies with Apoxin-I (apoptosis factor with LAAO activity isolated from *C. atrox* venom) suggest that losing its N-glycosylation is implicated in abolishing the catalytic activity of this toxin, consequently altering its biological activities, such as the induction of cellular apoptosis [39–41]. Izidoro et al. [9], on the other hand, suggested that the carbohydrates present in the structure of SV-LAAOs mainly play a structural role and protect the enzymes against proteolysis, since the venoms are rich in proteolytic enzymes. In addition, studies suggest that the glycan moiety is responsible for the anchoring of these enzymes in the host cell, which may explain the ease of LAAOs in directing hydrogen peroxide to the target cell, thereby inducing cellular apoptosis [12].

## Conclusions

In conclusion, our results demonstrate that both *Bothrops* LAAOs (BjussuLAAO-II and BmooLAAO-I) have a high affinity for hydrophobic amino acids, such as Phe and Leu. In addition, they maintain their enzymatic activities in a wide range of temperatures and pH values, also presenting high enzymatic stability for over a month when stored at 4 °C in a neutral pH solution. These results may guide new studies with SV-LAAOs, since the maintenance of enzymatic activity after long periods of storage is essential to allow not only their functional studies but also their biotechnological use.
